# Application of flat panel OLED display technology for the point-of-care detection of circulating cancer biomarkers

**DOI:** 10.1038/srep29057

**Published:** 2016-07-04

**Authors:** Benjamin A. Katchman, Joseph T. Smith, Uwadiae Obahiagbon, Sailaja Kesiraju, Yong-Kyun Lee, Barry O’Brien, Korhan Kaftanoglu, Jennifer Blain Christen, Karen S. Anderson

**Affiliations:** 1 Center for Personalized Diagnostics, Biodesign Institute at Arizona State University, Tempe, AZ 85281 USA; 2 Flexible Electronics and Display Center at Arizona State University, Tempe, AZ 85284 USA; 3 School of Electrical Engineering at Arizona State University, Tempe, AZ 85281 USA

## Abstract

Point-of-care molecular diagnostics can provide efficient and cost-effective medical care, and they have the potential to fundamentally change our approach to global health. However, most existing approaches are not scalable to include multiple biomarkers. As a solution, we have combined commercial flat panel OLED display technology with protein microarray technology to enable high-density fluorescent, programmable, multiplexed biorecognition in a compact and disposable configuration with clinical-level sensitivity. Our approach leverages advances in commercial display technology to reduce pre-functionalized biosensor substrate costs to pennies per cm^2^. Here, we demonstrate quantitative detection of IgG antibodies to multiple viral antigens in patient serum samples with detection limits for human IgG in the 10 pg/mL range. We also demonstrate multiplexed detection of antibodies to the HPV16 proteins E2, E6, and E7, which are circulating biomarkers for cervical as well as head and neck cancers.

There is an emerging recognition that rapid, effective molecular diagnostics can improve the efficiency of global healthcare delivery. Point-of-care molecular diagnostic devices have emerged to meet the need for providing health care that is more affordable and accessible, by replacing centralized medical diagnostic laboratory testing with simple to use, low-cost, disposable devices that analyze circulating biomarkers in blood, saliva or urine to quickly assess patient health^1^,^2^,^3^,^4^. Point-of-care diagnostics are particularly needed in low-to-middle income countries (LMICs) where centralized laboratory testing is cost-prohibitive and in many cases non-existent. Projections indicate that substituting centralized laboratory testing with point-of-care testing that can be done instead by the patient at home or by a healthcare worker with limited training in a non-clinical setting may be able to reduce total healthcare costs in these regions by as much as 66 percent, combined with improving overall healthcare delivery^5^,^6^,^7^. Recently, there has been a substantial effort in both the development and the availability of new rapid point-of-care diagnostic assays that address a variety of diseases and medical conditions^8^. To accurately diagnose and monitor patients prior to the onset of clinical symptoms, monitor for disease progression and recurrence, and/or to detect more complex diseases, such as cancer – which is the focus of this manuscript - the lower limit of detection for circulating biomarkers needs to be approximately 100 to 1000 times more sensitive, approaching pg’s/mL^9^. In addition to requiring a wide dynamic range and high sensitivity, the detection of circulating cancer biomarkers also requires the simultaneous analysis of multiple biomarkers in patient biofluid samples to improve both analytical sensitivity and specificity^8^,^9^.

Circulating tumor-specific antibodies have been identified as cancer-specific biomarkers for early detection as select antibodies that can be detected in cancer patients years prior to the onset of physical symptoms^10^,^11^,^12^. Of the tumor-specific antibodies, IgG antibodies (Abs) specific to the early antigens of human papillomavirus (HPV), are strongly associated with HPV-associated cancers, such as cervical, oropharyngeal, and anal cancers^10^,^13^,^14^,^15^,^16^,^17^. HPV16-specific Abs have been detected years prior to clinical diagnosis and represent potential circulating biomarkers for early detection. Clinically, the incidence of HPV-positive cancer infection has dramatically increased over the past three decades and is now recognized as an ongoing global health crisis with over 500,000 preventable new cases yearly worldwide^18^, despite evidence that HPV vaccination is safe and effective. However, existing HPV vaccines are often underused and/or unavailable to a large portion of the world^19^. In addition, screening for high-risk HPV nucleic acid associated with a high risk of developing cancer is currently cost prohibitive in LMICs emphasizing the need for a low-cost point-of-care diagnostic assay for the early detection of HPV positive patients^19^.

In our group, we have developed fluorescent protein planar microarrays for the high-throughput screening of circulating antibodies, including high-risk HPV type 16 that is directly associated with the progression of cervical and oropharyngeal head and neck cancer^10^,^13^. Fluorescent planar protein microarray technology combines high analyte sensitivity with multiplexed detection^20^,^21^,^22^. For analysis, each biorecognition site is actively interrogated with a bright light source and the emitted fluorescent signal is detected electronically. However, to date, high sensitivity, multiplexed fluorescent biorecognition planar microarray technology has not been translated from diagnostic laboratories into a low-cost, disposable point-of-care platform, in part because existing fluorescence measurement-based equipment typically requires large and expensive optical components such as optical microscopes (for magnifying optics), lasers, and low-light digital cameras, which are not compatible with low-cost and fully-disposable point-of-care configurations^8^. As a solution, our work has focused on miniaturizing these particular optical components into a simple and low-cost disposable configuration that is designed for both high sensitivity and multiplexed detection. In our approach, a small organic light emitting diode (OLED) array from the commercial flat panel display industry replaces the laser light source, and a photodiode array replaces the low light digital camera. The OLED display technology-based configuration is designed to eliminate the need for magnifying optics by sandwiching the fluorescent biorecognition layer directly between the OLED emitter and photodiode. This isn’t a new configuration^23^,^24^,^25^,^26^, but in the approach reported in this work, we address the previously reported inability to detect the fluorescent signal emitted from more than a single biomarker, along with resolving the observed poor diagnostic sensitivity in the previously reported OLED display-based biosensor configurations.

Here, we combined programmable high-density, fluorescent protein microarray technology developed at the Arizona State University (ASU) Center for Personalized Diagnostics^20^,^27^ with a small and very bright OLED flat panel display (array) developed at the ASU Flexible Electronics and Display Center (FEDC). The microarray and OLED array are integrated with optical filters in place of the crossed polarizers used in earlier reported work^21^,^22^,^28^,^29^,^30^,^31^ to fabricate a prototype point-of-care immunosensor with markedly improved analytical sensitivity. The active arrays enable multiplexed detection of multiple biomarkers in a single patient sample, while fluorescent biorecognition provides diagnostic laboratory level sensitivity when combined with optical interference filters and charge integration for signal readout. This configuration is designed to analyze patient blood samples in a multiplexed, disposable point-of-care device, and rapidly provide a quantitative result related to the biomarker being detected with high sensitivity. Leveraging commercial flat panel display technology offers a largely untapped opportunity to provide a fundamental breakthrough in reducing the cost to manufacture point-of-care biosensors as well as improve their functionality, while also using a well-established industrial base already capable of supplying a large number of consumer electronic products annually. For perspective, in 2012 flat panel displays were manufactured at a rate of 100 square kilometers per year^32^, which is enough material to completely cover the island of Manhattan. Despite extremely high capital equipment costs that can approach several billion dollars ($US), commercial flat panel display factories currently can manufacture displays for less than 10 cents per square centimeter, based on today’s HDTV pricing. Our previously reported work introduced the concept and compared the performance to a typical fluorescent laboratory enzyme linked immunosorbent assay (ELISA) plate reader^30^,^31^. In this new report, we present an improved prototype OLED display technology-based configuration that provides diagnostic sensitivity of human IgG at or below 10 pg/mL, which approaches the lower limit of detection for typical clinical laboratory instrumentation. We also demonstrate the serological detection of HPV 16 E7 circulating IgG antibody biomarker for HPV-associated head and neck cancer using the OLED-based test configuration, and provide a comparison to an established laboratory ELISA platform^10^,^13^. Finally, we demonstrate the multiplexed detection of IgG antibodies in sera from patients with head and neck cancer to the HPV 16 proteins E2, E6, and E7, using the same test configuration and contrast and compare the sensitivity and specificity of our new point-of-care test to our previously reported laboratory based assay.

## Results

### Adaptation of commercial OLED flat panel display technology for fluorescent-based biorecognition

**Figure 1:**
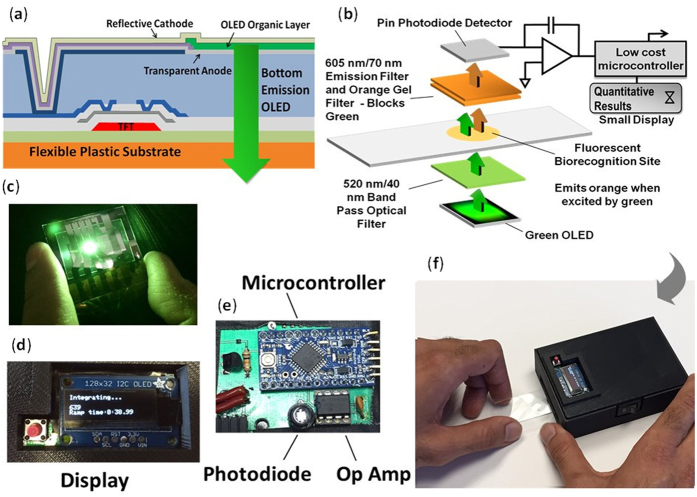
(**a**) A cross-section of the phosphorescent green OLED device structure in our display. (**b**) An exploded schematic view of our OLED display-based point-of-care test configuration, combining green OLED technology for optical excitation, photodiode, fluorescent biorecognition layer, and optical filters for high-sensitivity fluorescence analysis and detection of disease biomarkers. (**c**) A photograph of our 515 nm, 5 mm^2^ bottom emitting green OLED display test structure used for this study. (**d**) A small display used to provide instructions, status, or test results. (**e**) Our prototype electronics test board containing a microcontroller and Op Amp-based charge integrated readout circuit which provides a ramp time that inversely correlates with fluorescent biorecognition signal intensity. (**f**) Our hand-held 3D printed point-of-care prototype, which aligns the fluorescent biorecognition site on the microscope slide with the OLED emitter, face-to-face excitation and emission optical filters, biorecognition site, and photodiode.

As illustrated in Fig. 1b, our approach for point-of-care diagnostics sandwiches a fluorescent biorecognition site between a green OLED emitter and a photodiode. OLED displays are electroluminescent devices, commonly used today in smart phone color displays, which emit light when a forward voltage bias is applied across a transparent anode and reflective cathode. In operation and as illustrated in Fig. 1, the green OLED emitter is activated to illuminate the immobilized fluorescent biorecognition site. A 520 nm/40 nm bandpass optical excitation filter blocks any orange light emitted by the green OLED. Any illuminated fluorescent material captured on the biorecognition sites now re-emits longer wavelength orange light (for a green excite/orange emit fluorophore label). The emitted orange light then passes through the 605 nm/70 nm long-pass optical emission filter and orange gel filter, where it is detected by the silicon photodiode, while the shorter wavelength light from the green OLED is blocked by the optical filters. This planar face-to-face sandwich-style (i.e. focal plane) optics configuration is required to prevent the weak fluorescence signal from being swamped out by bright green light from the OLED emitter and is key in providing point-of-care diagnostic sensitivity that approaches the capabilities of a clinical laboratory. As shown in Fig. 1, an additional orange gel filter was placed between the 605 nm/70 nm emission filter and the photodiode detector. Even with the high performance optical emission filter, we still observed green light from the OLED reaching the photodetector. Adding the inexpensive orange gel filter reduced the background light level by approximately an additional factor of about 100 times, while only reducing the detectable fluorescent signal level by 10 to 20 percent. For multiplexed detection of biomarkers, we envision pairing multiple OLEDs in a flat panel display-based array with opposing pitch matched thin film photodiodes, with one OLED/photodiode pair for each biomarker recognition site.

We used a charge integrating operational amplifier (Op Amp) circuit to measure and quantitatively readout the detected fluorophore signal level from the detected biomarkers. As shown in the simplified electronics diagram in Fig. 1, this new readout approach uses a photodiode connected to a low-noise charge integration Op Amp circuit, which converts the detected charge from the photodiode to a relatively large output voltage that can be easily detected by a low-cost microcontroller. The photodiode is operated in photovoltaic mode (zero bias) to eliminate any effects from the photodiode dark current on the output signal. The 4 mm^2^ silicon photodiode was selected to provide comparable responsivity and dark current to the flexible amorphous silicon photodiode pixels used in our flexible digital x-ray detectors^30^,^33^,^34^,^35^ and approximately match the size of the green OLED emitter. To quantitatively measure the fluorescent signal, the time (in seconds) is measured as the detected signal ramps from 0 volts to the supply voltage rail of approximately 8.4 volts. The Op Amp is powered on and off to start and stop the voltage ramp, respectively, with the ramp time Δ*t* (sec) expressed by the following relationship:


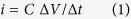


As described in equation 1, the fluorescent signal is reported as output voltage Δ*V*, which is stored by an integrating capacitor *C*. When more (orange emitting) fluorophores are captured on the antibody biorecognition site, the detected signal ramps faster and reaches the voltage rail in a shorter period of time due to the higher current *i* detected by the photodiode. For sample analysis, the ramp time Δt, is now inversely proportional to the concentration of the fluorescently labeled Ab biomarker in the patient sera. This provides the desired quantitative relationship between analyte concentration and the detected output. The primary advantage of this system is the ability to use long op-amp charge integration times (30 to 60 seconds) to detect extremely low light levels from a very small number of fluorophores captured on the biorecognition site, by providing a robust electrical signal (output voltage) to help separate out the detected and extremely weak fluorophore signal from the background noise level. The microcontroller then translates the detected voltage signal into quantitative information provided on a small integrated display (Fig. 1d). While previous research has evaluated using lock-in amplifier technology to detect the very low signal (light) levels from the excited fluorophores to improve sensitivity, we believe for very low-cost and ultimately disposable point-of-care applications, with a target cost of less than $10 (US), a much less costly electronics detection method is required. Initially, we evaluated a very high gain Op Amp-based transimpedance amplifier circuit, but we found the low picoamp signals at the lower fluorophore concentrations were challenging to detect using low-cost electronic components. As a solution, we recognized that for this particular application, real-time instantaneous detection was not necessary. Instead, we exploit a tradeoff between detection time and accuracy with a simple, low-cost charge integration Op Amp-based circuit, where longer integration time translates to higher sensitivity.

### High Intensity Green OLED Display Development

**Figure 2:**
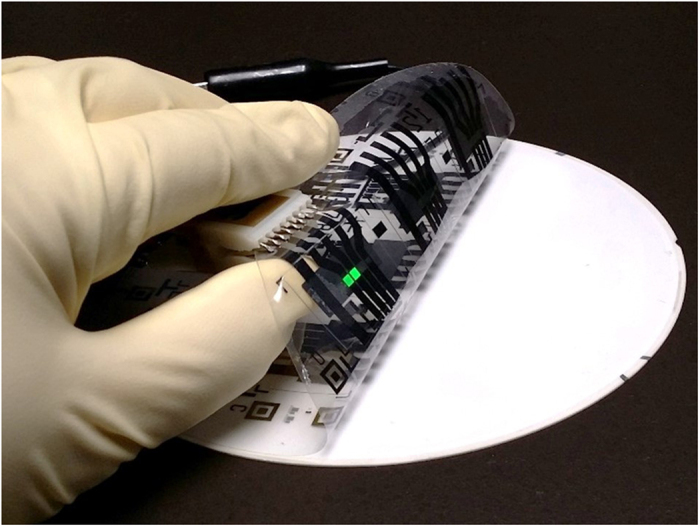
ASU Flexible green OLED test structures on 6″ plastic substrate manufactured using temporary bond/de-bond process on rigid substrate. After thin film processing, flexible OLEDs are simply peeled off.

The green OLED test structure used for this work, shown in Figs 1c and 2, was manufactured on a flexible plastic substrate, and has a peak emission intensity at 515 nm^28^,^35^. However, OLEDs of comparable brightness manufactured on conventional rigid glass substrates are expected to have similar performance. Conceptually, the approach used to make a display flexible is straightforward and is essentially identical to the process used to manufacture large commercial flat-panel displays on glass substrates. To make the device flexible, the starting glass substrate is replaced in our process flow with a 125 μm thick DuPont Teijin Films Teonex Polyethylene Naphthalate (PEN) flexible plastic substrate temporarily bonded to a rigid carrier. After the thin film process steps are completed, the flexible plastic PEN substrate with patterned nanoscale thin film layers on top is peeled off, similar to peeling off a Post-it brand note (Fig. 2). The temporary rigid carrier allows the flexible display to be processed using unmodified, off-the-shelf, thin-film semiconductor process tooling, designed for rigid glass substrates or silicon wafers. Additional details on the display process used to manufacture the green OLEDs used for this work can be found in O’Brien, *et al.*^28^ and in the methods section at the end of this report.

### Preliminary demonstration of fluorescent programmable immunoassay sensitivity and LLOD

**Figure 3:**
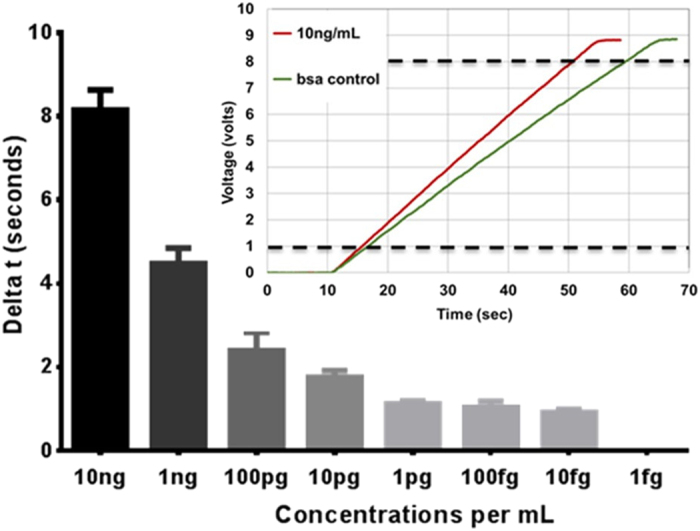
ELISA assay results for the detection of whole human IgG by fluorescent biorecognition. Dylight 549 anti-human fluorophores bound to the whole human IgG are excited by a green OLED and the emitted orange light is quantified. From the inset plot, the signal output is determined by the ramp time from 1 to 8 volts. The difference in ramp time (or time to reach threshold) between our sample and a BSA control quantifies concentration as *delta t*, demonstrating a conservative lower-limit-of-detection of 10 pg/mL.

To determine the lower limit of detection (LLOD) of our prototype OLED display-based point-of-care test configuration (Fig. 1), we immobilized whole human IgG on an aminosilane coated microscope slide at concentrations logarithmically scaled from 10 ng/mL to 1 fg/mL. We then probed with fluorescent anti-human Dylight549^®^ (556 nm excite/571 nm emit) and determined the ramp time for each concentration. However, instead of calculating the ramp time from 0 volts to the 8.4 V supply rail, we captured ramp times using the ramp time difference or delta between 1 and 8 volts (Fig. 3). This eliminated any effects from small variations in the battery supply voltage as well as any start time variations for the different concentrations. Additionally, we normalized all of our ramp times to a concentration matched bovine serum albumin (BSA) negative control by defining *delta t* as the difference between the BSA negative control ramp time and the IgG recombinant protein ramp time (Fig. 3). The *delta t* time (in seconds) directly corresponds to the captured fluorophore concentration on the biorecognition site that is above the background BSA control level. Hence, the biorecognition site is positive (i.e., a detection) when the *delta t* is greater than 0 seconds. For example, in Fig. 3, the recorded ramp time for the 10 ng/mL concentration was 8 seconds faster than the ramp time for BSA control. This is recorded as a *delta t* of 8 seconds and corresponds to a strong positive response. As expected (Fig. 3 bar chart), the observed *delta t* decreases as the IgG concentration is decreased, with a positive response (*delta t *> 0 seconds) observed all the way down to 10 fg/mL. However, below 10 pg/mL, we no longer observed a decrease in the *delta t,* so the LLOD in this preliminary work is set to 10 pg/mL.

Typical low-cost microcontrollers that could be used for signal readout in a point-of-care electronics configuration (Fig. 1) include an integrated 10-bit analog to digital converter (ADC) able to resolve ramp time differences down to ~39 milliseconds (40 seconds ramp time ÷2^10^). This implies that even lower circulating biomarker concentrations than 10 pg/mL could be resolved^8^.

### Demonstration of fluorescent immunoassay for the detection of serum HPV16 E7 antibodies

**Figure 4:**
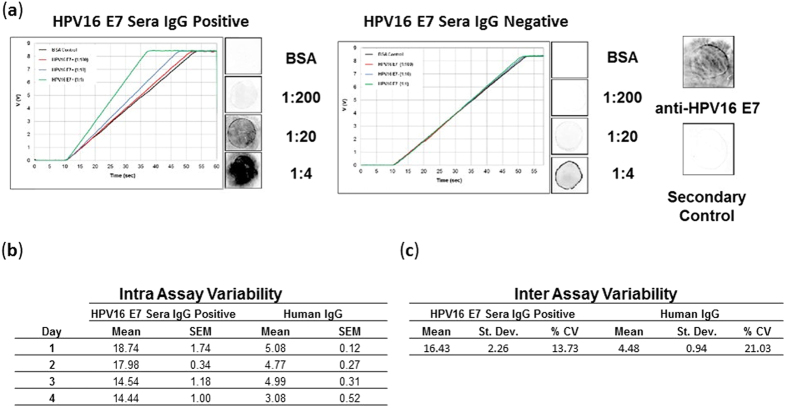
Detection of HPV antibodies in human sera. Using our test configuration we (**a**) immobilized recombinant HPV16 E7 protein and specifically detected HPV16 E7 antibodies from serum samples over a series of dilutions (1:4, 1:20, and 1:200). Representative images from each corresponding dilution as well as integrated ramp plots demonstrate assay specificity and background or off-target signal levels. To determine intra and inter assay variability we screened serum positive for HPV16 E7 antibodies and whole human IgG positive control in duplicate over four consecutive days. (**b**) Calculated values of intra assay standard error of the mean and (**c**) inter assay coefficient of variance.

HPV infection is the most commonly diagnosed sexually transmitted disease in the United States^36^. Infection with high-risk HPV is necessary for cervical cancers (99.7%) as well as the majority of oropharyngeal head and neck squamous cell carcinoma (65–80%)^18^. While screening by a combination of cytology and high-risk HPV typing has markedly decreased cervical cancer incidence in developed countries, there is a need for accurate and low-cost point-of-care assays for the biologic changes that are associated with progression of HPV infection to HPV cancer^36^. Previously, we and others have reported that HPV16 E2, E6, and E7 IgG antibodies are specifically detected in the sera of patients with HPV-associated cancers^10^,^13^,^17^,^37^,^38^. Our Center has developed the Rapid Antigenic Protein *in Situ* Display (RAPID) ELISA, which is a programmable ELISA assay in 96-well plates that can be readily adapted to measure antibodies to any *in vitro* expressed tagged antigen^10^,^21^,^22^. However, this assay requires a protein display biochemistry, luminescent detection, highly-trained personnel, and expensive detection equipment that is not feasible as a low-cost point-of-care diagnostic assay. Here, we have adapted this immunoassay for the serological detection of HPV16 E7 antibodies using OLED-based fluorescent detection of human IgG. For this study we procured serum samples from the HOTSPOT biorepository, each sample was tested by PCR for the presence of HPV16 DNA^14^. To demonstrate the accuracy, specificity, and inter- and intra-assay reproducibility of the optical configuration, we generated full-length recombinant HPV16 E7 protein and manually spotted pitch-matched HPV16 E7, BSA and whole-human IgG protein onto aminosilane coated glass microscope slides. The slides were blocked in 5% milk in 0.2% PBS-Tween20 followed by incubation with patient sera. We then determined the presence of IgG antibodies against HPV16 E7 by probing the slide with fluorescent anti-human IgG Dylight549 antibody and measured the relative fluorescence. In our preliminary configuration our total assay time is two hours. Using monoclonal antibodies against HPV16 E7, the immobilization of HPV16 E7 recombinant protein was confirmed (Fig. 4a, anti-HPV16 E7). We immobilized 25 ug/mL of recombinant HPV16 E7 protein and determined the optimal signal-to-noise ratio by diluting serum from a previously identified subject with high titers of IgG antibodies specific for HPV16 E7 (HPV16 E7 IgG Positive) and serum from a healthy control (HPV16 E7 IgG Negative) (Fig. 4a). Dilutions of sera at 1:4, 1:40, and 1:200 in 5% milk-PBST were added and introduced into the detector which determined the delta t (sec) value for replicated samples. As illustrated in Fig 4a,b, day 1, even at a 1:4 dilution (comparable to the volume from a finger-prick point-of-care immunoassay) we observe only low levels of off-target fluorescent signal. We replicated the assay using the same sera at a 1:4 dilution over four consecutive days, and calculated the intra-assay variability represented by the standard error of the mean in duplicate samples each day. The intra-assay variability remained low and is representative of current standards for diagnostic assays (Fig. 4b). The inter-assay variability is shown as the coefficient of variance (CV) over the four-day period (Fig. 3c). Current analytic targets for inter-assay variability are below a CV value of 20%^39^,^40^. The observed CV values were 13% for E7-specific IgG antibodies. We have observed that variations in inter- and intra-assay variability are often introduced in the printing process. We expect that transitioning the printing process from manual spotting to a contact or piezo printer at high batch volume will reduce spot size variability and further improve inter- and intra-assay variability.

### Multiplexed detection of serum HPV16 E2, E6, and E7 antibodies

**Figure 5:**
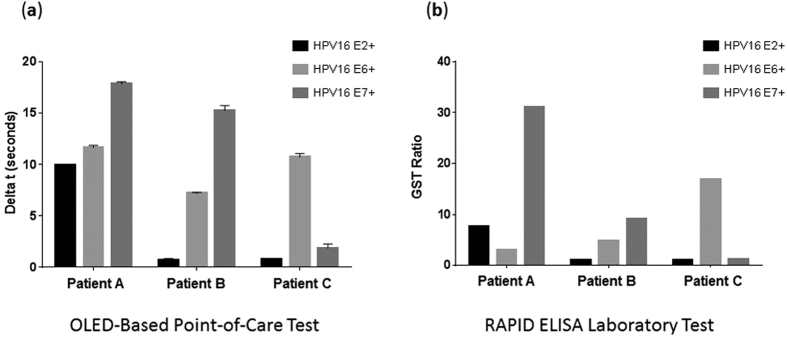
Multiplexed detection of human IgG in patient sera. To evaluate the detection of multiple biorecognition sites we immobilized recombinant HPV16 E2, E6 and E7 protein as well as human IgG and BSA at 6 mm intervals. We selected serum from three patients with known serum antibodies to all or one of the HPV16 proteins and (**a**) compared the results of our OLED-based point-of-care test configuration against our previously reported (**b**) RAPID ELISA assay measuring human IgG in the same serum samples. Results demonstrated comparable detection results to multiple serum antibodies for each of the three patients using our prototype test configuration to the RAPID ELISA diagnostic laboratory test.

In our previous study, we analyzed the utility of a multi-antigenic assay for the detection of patients with HPV16 positive oropharyngeal head and neck cancer^10^,^13^,^14^,^37^. The majority of patients were positive for HPV16 E1, E2, E6, and/or E7 antibodies. A case was determined positive by the presence of one or more positive antibodies. In this study, over 5% of patients with oropharyngeal head and neck cancer have antibodies to HPV16 E1 or E2, but no E6 or E7 antibodies. Using a multiparametric algorithm the sensitivity approaches 88% at 96% specificity. This supports the rationale for a multi-antigenic assay for the early detection of HPV positive oropharyngeal head and neck cancer. To demonstrate the preliminary ability of our OLED-based point-of-care biosensor configuration to detect multiple biorecognition sites, we immobilized 25 μg/mL of recombinant HPV16 E2, E6, E7, whole human IgG, and BSA on five separate ~2 mm diameter biorecognition sites spaced at 6 mm intervals on the same microscope slide (Fig. 5) and compared our point-of-care test configuration results with our RAPID ELISA laboratory test results. The five sites were detected using the Fig. 1 singleplex prototype test configuration by simply incrementing (pulling) the slide 6 mm out at a time to sequentially line up the sites with the center of the ~2 mm green OLED emitter. This approach provided a simple, but effective early demonstration of multiplexing performance using just a single emitter. For the multiplexed comparison, we selected three sera from OPC patients, previously identified through our custom RAPID ELISA assay (Fig. 5b) as being positive for IgG antibodies against HPV16 E2 and E7 (Patient A), HPV16 E6 and E7 (Patient B), and HPV16 E6 (Patient C) proteins. As illustrated in Fig. 5a,b, we detected multiple serum antibodies for each of the three patients using our prototype OLED-based point-of-care test configuration, comparable to the RAPID ELISA laboratory test. We observed increased detection of the HPV16 E6 antibody in patient 57 using our OLED-based point-of-care test configuration compared to our previously reported RAPID ELISA laboratory test that is likely due to differences in antigen display between the two assays. This demonstrated that the OLED-based point-of-care test configuration can be used to specifically detect individual bound IgG without evidence of cross-over interference, as well as provide comparable detection results to the RAPID ELISA laboratory test to multiple serum antibodies.

**Figure 6:**
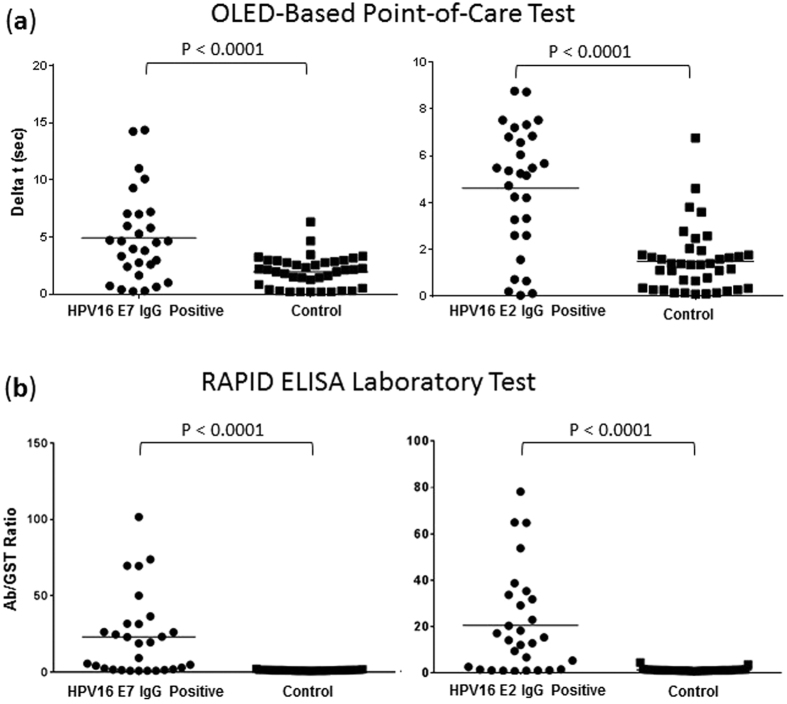
Comparison of OLED POC assay to Rapid ELISA laboratory test for multiplexed detection of human IgG in patient sera. Sera from 29 HPV16+ patients and 39 healthy controls were evaluated for the detection of HPV16 E2 and E7 specific IgG. The results of the (**a**) OLED-based point-of-care test configuration against our previously reported (**b**) RAPID ELISA assay are shown. OLED-based configuration demonstrated sensitivity of 59% at 95% specificity using a cutoff value of the mean of the controls +2 standard deviations compared to our previously reported RAPID ELISA laboratory test instrumentation with a sensitivity of 83% at 95% specificity. Significance was determined using a student’s t-test.

To determine the accuracy and specificity of the HPV16 multiplexed immunoassay, we immobilized 25 ug/mL of recombinant HPV16 E7, E2, whole human IgG, and BSA on five separate ~2 mm diameter biorecognition sites spaced at 6 mm intervals on a glass microscope slide. We randomly selected 29 HPV16+ serum samples as well as 39 healthy controls from our previously reported HOTSPOT study and screened them in duplicate on individual slides^14^. The 5 sites were detected as described above by incrementally pulling out the slide 6 mm at a time to line up the sites with the center of the green OLED emitter. We determined a cutoff value as the mean of the controls +2 standard deviations for each HPV antigen (OLED-Based Point-of-Care Test, HPV16 E7, 4.68; E2, 4.25; RAPID ELISA, HPV16 E7, 1.65; E2, 2.66). Using this cutoff value, the overall sensitivity and specificity defined as a case or control being positive for one or more HPV antigen (HPV16 E2 and/or E7) was 58% sensitivity at 95% specificity for the OLED-Based Point-of-Care test compared to 83% sensitivity at 95% specificity for the RAPID ELISA laboratory test (Fig. 6). The results described are comparable for HPV16 E2 and E7 to our RAPID ELISA laboratory test.

## Discussion

In this report, we have demonstrated the novel combination of commercial flat panel OLED display technology with high density fluorescent biorecognition microarray technology to fabricate a prototype point-of-care immunoassay. Fluorescence-based detection in point-of-care molecular diagnostics offers a number of both advantages and disadvantages^41^,^42^. The key advantages demonstrated in our reported results include 1) a wide dynamic range and sensitivity, due to the charged-based integration of the fluorescence signal and 2) a methodology for multiplexed immunoassays with a clear path to high volume manufacturing, grounded in two fundamental technologies: protein microarrays and OLED flat panel displays.

In comparison to a well-established laboratory ELISA, we have also demonstrated a similar accuracy and specificity in the detection of serum antibodies against HPV16 E7, a potential blood-based biomarker under evaluation for cervical and oropharyngeal head and neck cancer detection. Further, we present data supporting the multiplexed detection of serum antibodies against HPV16 E2, E6, and E7 with minimal signal interference. The recognized disadvantage of fluorescent biorecognition is the need for additional optics and electronics for detection, unlike conventional colorimetry-based lateral flow assays, which can be interpreted visually^43^,^44^,^45^. To mitigate this disadvantage, we developed a simplified point-of-care configuration using planar face-to-face optics that eliminated the need for separate magnifying optics by sandwiching the fluorescent biorecognition layer directly between an organic light emitting layer and photodiode. This was combined with optical filters and low-cost charge integration electronics to increase both sensitivity and dynamic range and decrease cost.

Point-of-care devices must leverage engineered signal enhancement techniques to approach the performance of large, expensive laboratory equipment in a portable, inexpensive configuration^42^. Lateral flow assays (LFA)s have emerged as the predominant point-of-care approach for biofluid samples^1^,^43^,^44^,^45^,^46^,^47^,^48^,^49^,^50^,^51^. These microfluidics devices, usually paper-based assays, enhance the signal detection relative to lab-on-chip or microscope slide assays by increasing the surface area of a porous media^1^,^43^,^45^,^48^. Many materials, such as nanoparticles, beads, and quantum dots, have been used to enhance sensitivity^45^,^46^. Alternative detection mechanisms, such as plasmonic^52^ and electrochemical^51^ also enhance signal detection^51^,^52^. Combinations of these techniques may improve the lower limit of detection by up to three orders of magnitude, in the pg/mL range^49^,^52^,^53^. Many of these techniques for signal enhancement are predicted to be compatible with our device.

Point-of-care medical diagnostic sensors using molecular biomarkers for mobile health have the potential to reduce healthcare costs by providing rapid feedback on disease states^42^. Advances in wearable and flexible sensor development, specifically in OLED and organic photodetectors provide a novel avenue to combine fluorescent planar array technology to provide a simple, highly-sensitive diagnostic device. While the work in this manuscript has focused on the detection of antibodies specific for HPV proteins, the programmable molecular and electronic configuration presented provides many opportunities to adapt this system to meet the demands of multiple types of analytes and clinical applications.

## Methods

### Serum samples

Serum samples were previously obtained at the time of clinical diagnosis from patients with newly-diagnosed HPV+ oropharyngeal cancer and healthy controls and the serologic responses to HPV16 have been previously reported^14^,^37^ (Clinical Trials number: NCT01342978). Written informed consent was obtained from all subjects in accordance with Arizona State University institutional review board approval. All experimental protocols were approved and were carried out in accordance with the relevant guidelines under the Arizona State University institutional review board. All of the methods involving human subjects were carried out in accordance with the relevant guidelines previously stated in ref. 14.

### HPV antibody detection in blood sample by RAPID ELISA

Serum samples (1:100) were tested for HPV16 E2, E6 and E7 IgG antibodies by programmable RAPID ELISA as previously described^21^. Proteins were expressed using a human HeLa cell lysate *in vitro* transcription and translation system (Thermo Scientific) and blocked with 10% Escherchia coli (E. coli) lysate. Luminescence was measured as Relative Light Units (RLU) as a ratio to GST-antigen control. Cut-off values for positive serology are defined as the mean +3 standard deviations of the RLU ratio observed among the healthy controls.

### Expression and purification of HPV16 E2, E6, and E7

pDEST15 (Life Technologies) was used to generate N- terminal Glutathione transferase fusion proteins. E2 protein was subcloned as the C-terminal fragment (CE2) for optimal protein expression^10^. E6, E7 and CE2 genes in the vector pDONR221 were transferred to the destination vector pDEST15 by recombination cloning and transformed into the BL21DE3 Ecoli strain. Isolated colonies were grown in LB media for 6–8 hr at 37 °C. The cultures were then diluted 1:20 into LB media and grown at 37 °C until OD600 of 0.6 was reached and induced with IPTG at 18 °C for 21 hr. After 21 h incubation at 18 °C, the bacteria were centrifuged at 5000 X g for 20 min at 4 °C, and the pellets were resuspended in lysis buffer (50 mM Potassium phosphate, pH 7.8, 400 mM NaCl, 100 mM KCl, IGEPAL 0.01%, 1 mM DTT, 25 μg/ml DNase, 2 mg/ml Lysozyme, 5 mM MgSO_4_, 100 μM PMSF). The lysate was frozen at −20 °C, then thawed to RT and mixed for 1 hr at 37 °C. The lysate was centrifuged at 5000Xg for 20 min at 4 °C and the supernatant was removed and mixed with Glutathione Sepharose 4 fast flow medium and allowed to bind O/N at 4 °C. Glutathione Sepharose medium was washed seven times with PBS pH 7.3 by centrifugation at 500Xg for 5 min. Elution buffer (Tris HCl pH 8 containing 20 mM GSH) was used to elute GST tagged proteins and a Bradford assay was used to quantitate the protein. Purity of proteins was determined by Sodium dodecyl sulfate (SDS) Poly acrylamide gel electrophoresis (PAGE).

### Fluorescent detection of human serum IgG using a point-of-care configuration

As illustrated in ref. 10, glass microscope slides (VWR International) were coated in a 2% aminosilane coating solution (Pierce) for 15 minutes at room temperature. The slides were then rinsed with acetone followed by distilled water and dried with filtered compressed air. Purified recombinant protein was diluted in distilled water to 25 ug/mL and spotted (5 μl) on the aminosilanated glass slides and allowed to dry at room temperature. As a control, BSA was diluted in distilled water to 25 ug/mL and spotted (5 μl) on separate aminosilanated glass slides and allowed to dry at room temperature. The protein spotting procedure was repeated four times. The slides were stored at 4 °C overnight. Slides were washed once in phosphate buffered saline pH 7.4−0.2% Tween20 (PBST) solution and blocked at room temperature in 5% milk-PBST for one hour. During the blocking step the serum samples were diluted 1:1 in a 5% milk-PBST and allowed incubate at room temperature. The slides were then removed from the blocking solution and incubated with the serum for 1 hour at room temperature and then rinsed once in PBST. The presence of human IgG antibodies specific for the recombinant proteins was detected using Dylight 549-conjugated AffiniPure Goat Anti-Human IgG (Jackson ImmunoResearch Laboratories, Inc.). As a positive control we probed the purified HPV16 E7 recombinant protein with a mouse monoclonal antibody to HPV16 E7 (Santa Cruz) and detected it with anti-mouse IgG AlexaFluor 555 (Life Technologies).

### Statistical considerations

OLED based point-of-care and RAPID ELISA laboratory analysis were performed in duplicate. Significant differences (*p*-value) in cases and controls were assessed by two-tailed student t-test. To assess the value of AAb to discriminate cases from controls we defined the cut-off value as the mean of the controls +3 standard deviations.

### OLED fabrication, characterization, and operation

The OLED devices used in this work were fabricated at the ASU Flexible Electronics and Display Center (FEDC) using patterned indium-tin oxide (ITO) on flexible substrates. The green OLED device structure has a peak emission intensity at 515 nm and consisted of a 10 nm layer of hexaazatriphenylene hexacarbonitrile (HAT-CN) hole injection layer (HIL), followed by a 40 nm 4-4′-bis[N-(1-naphthyl)-N-phenyl-amino]biphenyl (NPD) hole transport layer (HTL). A 10–50 nm green emissive layer (EML) was deposited onto the HTL and comprised a co-host structure of [Host1:Host2:Green dopant]. Next, a 10–30 nm thick hole blocking layer (HBL) was deposited onto the EML, followed by a 30 nm electron transport layer (ETL) consisting of doped 8-hydroxyquinolinolato-lithium (Liq). A 2 nm Liq electron injection layer (EIL) was deposited onto the ETL, followed by a 100 nm layer of MgAg or Al cathode metal. Device area was 0.05 cm^2^. Films were deposited by vacuum thermal evaporation in a Sunicel Plus 400 system made by Sunic Systems (Suwon, South Korea) at pressures below 5 × 10^−7^ Torr. Films were patterned using metal shadow masks with no break in vacuum between layers. The devices were encapsulated using a thin-film barrier material from 3 M Company. HAT-CN was purchased from Lumtec (Hsin-Chu, Taiwan), and the other organic materials were supplied by Universal Display Corporation (New Jersey, USA).

Encapsulated devices were tested in ambient conditions. Current density/voltage, radiance and electroluminescence measurements were made using a Keithley 2400 source meter, calibrated Si photodiode model 818-UV from Newport (Irvine, CA, USA), and Ocean Optics HR-4000 spectrometer, using Spectra Suite software (Ocean Optics, Dunedin, FL, USA). For all reported test results, the OLED was pulsed at 6 Hz, with an 8.8 volt forward bias, providing an instantaneous optical output power of 0.8 mW. Similar to a semiconductor diode, increasing the direct current (DC) bias voltage exponentially increases current, which results in the OLED light intensity to also increase exponentially. However, we observed that increasing the DC bias above 7 volts to obtain higher light intensity started to degrade the OLEDs organic emissive layers due to current induced, localized joule heating in the OLED organic layers^35^,^47^. As a solution, we found that pulsing the OLED power supply at less than 10 Hz, combined with applying a thin conformal metal foil heat sink to the cathode, the OLED operating voltage could be increased above 7 volts to subsequently increase the instantaneous light intensity without degrading or damaging the OLED organic layers. Essentially, pulsing the OLED at a low frequency appears to give the organic layers in the OLED a chance to recover and cool down before applying a voltage bias in the next period.

## Additional Information

**How to cite this article**: Katchman, B. A. *et al.* Application of flat panel OLED display technology for the point-of-care detection of circulating cancer biomarkers. *Sci. Rep.* **6**, 29057; doi: 10.1038/srep29057 (2016).
